# Geographic Distributions in Hypertension Diagnosis, Measurement, Prevalence, Awareness, Treatment and Control Rates among Middle-aged and Older Adults in China

**DOI:** 10.1038/srep37020

**Published:** 2016-11-14

**Authors:** Minghui Yin, Balekouzou Augustin, Zhen Fu, Mingming Yan, Adan Fu, Ping Yin

**Affiliations:** 1 Department of Epidemiology and Biostatistics, School of Public Health, Tongji Medical College, Huazhong University of Science and Technology, Wuhan, Hubei, China; 2 Department of Nursing, The Central Hospital of Wuhan, Tongji Medical College, Huazhong University of Science and Technology, Wuhan, Hubei, China

## Abstract

Hypertension is of public health importance in China, but information on geographic distribution on hypertension by map visualization is limited for middle-aged and older adults. Regional geographic variations remain unexplained. Our study is to present geographic distributions at the provincial level and identify provinces and municipalities with high hypertension diagnosis, measurement and prevalence rates and/or low awareness, treatment, control rates among aged 45+ adults in China. We used data collected from the China Health and Retirement Longitudinal Study (n = 13,583) of Chinese people aged 45 years or older. We used weighted rates for our analysis. The rates by provinces and municipalities were compared using map visualization, and explore the main factors of the disparity using ordinal logistic regression. Higher hypertension prevalence rates (56.3%) but lower hypertension awareness, treatment and control rates (37.3%, 21.1% and 14.9%, respectively) were observed in Guizhou. Shanghai and Beijing had the highest hypertension prevalence, awareness and treatment rates (65.0%, 87.8% and 80.0% for Shanghai, 57.5%, 88.6% and 77.5% for Beijing, respectively). Remarkable variations were observed among surveyed provinces and municipalities. Several Chinese regions show particularly higher prevalence rates and/or lack of hypertension awareness and poor control.

Cardiovascular and other non-communicable diseases are currently responsible for two thirds of global mortality^1^,^2^, and cardiovascular disease is still the leading cause of death in China^3^. It is also very common, its global prevalence being about 40%^2^. Hypertension is a consistent and independent risk factor for cardiovascular and kidney diseases and stroke^4^,^5^,^6^,^7^. Hypertension plays a part in approximately 55% of the global mortality caused by cardiovascular diseases and in 7% of all disability-adjusted life years^8^. It has been estimated that hypertension was associated with 20% of the deaths recorded in China in 2005, including 2.33 million, nearly 80%, of the deaths from cardiovascular disease^9^. In China, the overall prevalence of hypertension rose substantially between 2002 and 2010–from around 20% to 34%^10^,^11^. Unfortunately, the management of hypertension in China has been ineffective for many years. For example, in 2010, only 35.7% of hypertensive individuals were aware of their actual conditions and fewer than 18% of such individuals were effectively controlling their hypertension^11^,^12^,^13^,^14^. Especially, the management of hypertension in elderly is a significant problem^15^,^16^.

In many survey studies with a component or a focus on health, prevalence of hypertension is according to “self-reports”: respondents are asked to report whether they have hypertension at present and/or have ever been diagnosed with hypertension. However, the actual prevalence, based on self-reports, might be seriously underestimated. This is because moderate, and even high levels of hypertension are typically asymptomatic and the hypertension awareness rates is generally low^17^. An effective way to assess the extent for hypertension awareness is to collect both objective measures and self-reported for the same respondents. This is, however, rarely done in survey studies. China Health and Retirement Longitudinal Study (CHARLS) collected both of the information. In addition, antihypertensive medication for the respondents was also included in CHARLS.

A few studies reported hypertension prevalence, awareness, treatment and control rates in China^12^,^18^,^19^, but few regional reports have revealed intriguing distribution on hypertension among middle-aged and older adults in China by map visualization. Regional geographic variations remain unexplained.

Here we presented geographic distributions at the provincial level and identify provinces and municipalities with high hypertension diagnosis, measurement and prevalence rates and/or low awareness, treatment, control rates among aged middle-aged and older adults across 28 provinces and municipalities in mainland china using China Health and Retirement Longitudinal Study (CHARLS) 2011 first wave data by map visualization, and explore the main factors of the disparity using ordinal logistic regression. We identified several regions of great concern.

## Methods

### Data

CHARLS, harmonized with the U.S. Health and Retirement Study (HRS) family of surveys, study on ageing, is publicly available and de-identified. The participants aged 45 years or older. The data were collected in a survey in which four-stage, stratified, cluster sampling was used to select eligible individuals. 150 county-level units from 28 provinces and municipalities, excluding Tibet, Ningxia and Hainan in mainland China, were selected to give a mix of urban and rural settings and a wide variation in the level of economic development. A structured questionnaire with several main sections was used to collect data from each respondent. The participants completed a computer-assisted personal interview (CAPI) in their home. As explained in detail by Zhao *et al.*^20^,^21^.

For our analysis, we used the first wave (2011) of CHARLS which was conducted between June 2011 and March 2012. Information on individual attributes, socioeconomic characteristics and urban/rural settings were collected. Blood pressure measurements were also collected.

### Hypertension diagnosed, measured, prevalence, awareness, treatment and control

#### Diagnosed

CHARLS collected information on individual self-reports of specific conditions with the general question: “Have you been diagnosed with hypertension by a doctor?”. We classified respondents as having “diagnosed hypertension” if they reported having hypertension.

#### Measured

Measured of hypertension was defined as hypertensive if their systolic blood pressure (SBP) ≥ 140 mmHg and/or diastolic blood pressure (DBP) ≥ 90 mmHg^22^,^23^. The key advantage in using data collected in CHARLS is that blood pressure is measured in the survey. Details on how blood pressure measurements are taken in the survey can be found elsewhere^20^. Briefly, in CHARLS, each respondent’s systolic and diastolic blood pressures were recorded three times by a trained and qualified nurse using an HEM-7112 electronic monitor (Omron, Kyoto, Japan) in the respondent’s home. We used a binary variable for measured hypertension according to the mean value of the three readings.

#### Prevalence

The prevalence was defined as hypertensive if either they self-reported to be hypertensive and/or have a blood pressure value above the diagnostic threshold, which is the mean SBP ≥ 140 mmHg and/or mean DBP ≥ 90 mmHg.

#### Awareness

Awareness of hypertension was defined as participants having been informed at least once by a health care professional that they had hypertension.

#### Treatment

Hypertensive patients were categorized as treatment if they were taking a prescribed medicine for management of hypertension.

#### Control

Control of hypertension was defined as antihypertensive treatment associated with mean SBP < 140 mm Hg and mean DBP < 90 mm Hg.

CHARLS also collected information on several health-related behaviors. We identified two categories for marital status: Married or cohabiting and Unmarried or single. We classified level of education as illiterate, primary education, secondary education and at least college level based on Chinese education system. We use BMI to identify whether respondents are normal (BMI < 25), overweight (BMI ≥ 25 and < 30) and obese (BMI ≥ 30). We identified three categories for smoking: current smoker, past smoker and never smoked according to the respondents’ tobacco use answer; and two categories for drinking: current drinker and abstainer according to their alcohol consumption answer.

As for the income, we calculated the household income based on the sub-questionnaire of household income according to all household members. As for the income, we employed information from a single comprehensive question about income according to all household members. We corrected household income via dividing it by the square root of the number of persons in the household^24^,^25^,^26^. Then, we assigned individuals into corresponding income terciles: first tertile (low income), second tercile (middle income) and third tercile (high income)^27^. CHARLS 2011 data file is available at: http://charls.ccer.edu.cn/en/page/data/2011-charls-wave1.

## Statistical Methods

### Map Visualization

In our analysis, we use weighted rates on hypertension to present geographic distributions for 28 provinces and municipalities in China. CHARLS released the weights, which take account of the national representativeness of the results and the missing anthropometric measurements^21^. Rates by provinces and municipalities were compared using map visualization^28^,^29^. Spatial data for administrative provinces were collected from National Geomatics Center of China^30^.

### Ordinal Logistic Regression

Based on diagnosed, measured, prevalence, awareness, treatment and control of hypertension we defined, we divided each category of rates into quintiles. (i.e., the respondents were split into five groups of similar size at 20%, 40%, 60%, and 80% after ranking all the provinces and municipalities by each category of rates, respectively). Subsequently, ordinal logistic regression with a Logit link was used to analyze the association between the multiple risk factors and quintiles of the prevalence rates of hypertension and quintiles of the control rates of hypertension, respectively. (Model 1: multiple risk factors and quintiles of the prevalence rates of hypertension; Model 2: multiple risk factors and quintiles of the control rates of hypertension). Ordinal logistic regression was chosen because quintiles classification consisted five categories, with increasing values (0–20% quintile = 1, 20–40% quintile = 2, 40–60% quintile = 3, 60–80% quintile = 4, 80–100% quintile = 5). The prevalence rates of hypertension were described as mild, mild-moderate, moderate, moderately severe or severe according to the categories from 1 to 5. The control rates of hypertension were classified to poor, fair, average, good or excellent control according to the categories from 1 to 5. Ordinal logistic regression was used to estimate the odds ratio (OR) and its 95% confidence interval. The level of statistical significance was set at 0.05.

The data were analyzed using R version 3.2.5 (R Core Team 2016, Vienna, Austria)^31^, map visualized by “maptools”^32^ and “ggplot2”^33^ packages, and ordinal logistic regression by “MASS” package^34^.

## Results

### Baseline characteristics

We summarized the baseline characteristics, both unweighted and weighted values, of the 13,583 respondents who were included in our analysis (Table 1). The weighted rates were similar to the unweighted values. Weighted hypertension of diagnosed, measured, prevalence, awareness, treatment and control rates were 24.2%, 31.9%, 41.7%, 57.9%, 46.4% and 23.4%, respectively.

### Hypertension diagnosis, measurement, prevalence, awareness, treatment and control rates by quintiles

We reported both weighted and unweighted hypertension diagnosis, measurement, prevalence, awareness, treatment and control rates for surveyed provinces and municipalities, separately (Tables 2 and 3). The weighted rates were also similar to the unweighted values.

**Figure 1:**
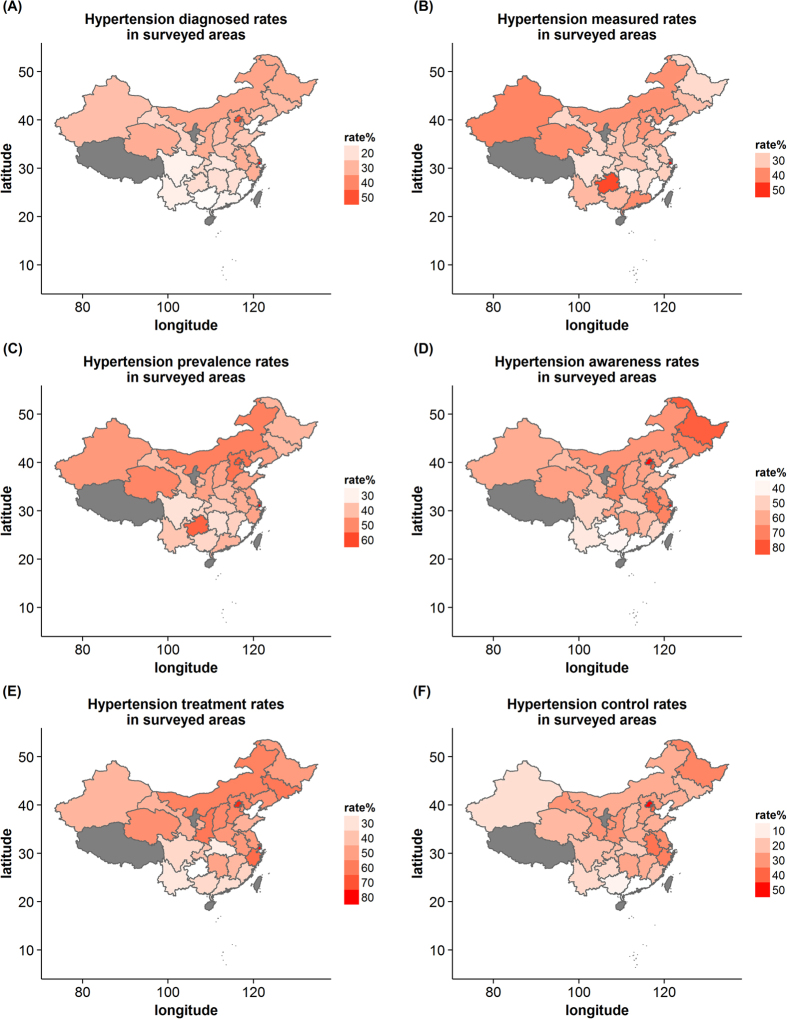
The hypertension diagnosis, measurement, prevalence, awareness, treatment and control rates for surveyed provinces and municipalities by maps, respectively. (The maps were generated using R version 3.2.5, https://www.r-project.org/).

**Figure 2:**
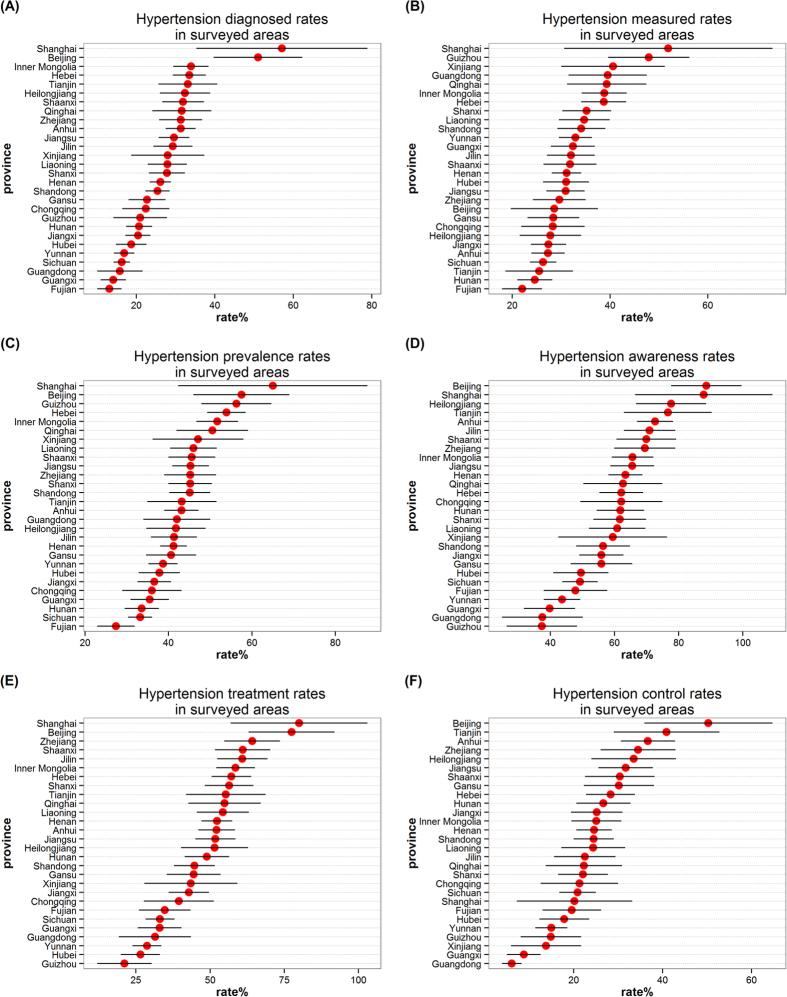
The hypertension diagnosis, measurement, prevalence, awareness, treatment and control rates for surveyed provinces and municipalities by dot plots, respectively.

The map was plotted using weighted rates (Fig. 1). Provinces and municipalities with available data were colored from white to red according to rates values; provinces and municipalities in grey have no data available. Also, we used dot plots to show weighted rates for provinces and municipalities (Fig. 2).

For hypertension diagnosis, the top 20% rates were Shanghai, Beijing, Inner Mongolia, Hebei, Tianjin and Heilongjiang, and the rates, in descending order, were 57.1%, 51.0%, 33.9%, 33.5%, 33.1% and 32.4%, respectively.

For blood pressure measurement, the top 20% rates were Shanghai, Guizhou, Xinjiang, Guangdong, Qinghai and Inner Mongolia, and the rates were 51.9%, 47.9%, 40.6%, 39.5%, 39.3% and 38.8%, respectively.

For hypertension prevalence, the top 20% rates were Shanghai, Beijing, Guizhou, Hebei, Inner Mongolia and Qinghai, and the rates were 65.0%, 57.5%, 56.3%, 53.9%, 51.7% and 50.5%, respectively. We also noticed the lowest rates was in Fujian (27.4%).

For hypertension awareness, the bottom 20% rates were Guizhou, Guangdong, Guangxi, Yunnan, Fujian and Sichuan, and the rates, in ascending order, were 37.3%, 37.5%, 39.7%, 43.6%, 47.8% and 49.2%, respectively.

For hypertension treatment, the bottom 20% rates were Guizhou, Hubei, Yunnan, Guangdong, Guangxi and Sichuan, and the rates were 21.1%, 26.5%, 28.7%, 31.4%, 33.0% and 33.1%, respectively.

For hypertension control, the bottom 20% rates were Guangdong, Guangxi, Xinjiang, Guizhou, Yunnan and Hubei, and the rates were 6.1%, 8.8%, 13.8%, 14.9%, 15.0% and 17.9%, respectively.

Remarkable variations were observed among surveyed provinces and municipalities.

### Ordinal Logistic Regression

The results of ordinal logistic regression are shown in Tables 4 and 5. Both of the models presented a good fit (p < 0.001), and the test of parallel line could not reject the null hypothesis (p > 0.05).

Significant factors of the prevalence of hypertension were overweight or obesity, smoking, drinking and education. From education, the coefficients estimates changed from negatively statistical significant to positively, showing that this association between the prevalence of hypertension and the level of education converted from negative to positive. For household income, the estimate for the third tercile (coefficient estimates = 0.118, p = 0.003) is significantly different from zero, showing that this value is associated with higher values on the prevalence of hypertension. We see that coefficients estimates of tercile tend to increase, although the second tercile is not sufficient to say that it is different from zero.

Significant factors of the hypertension control were age, marital status, overweight or obesity. From education, the coefficients estimates converted from negatively statistical significant to positively, showing that this association between the control of hypertension and the level of education converted from negative to positive. For household income, the estimate for the third tercile (coefficient estimates = 0.345, p < 0.001) is significantly different from zero, showing that this value is associated with higher values on the hypertension control.

## Discussion

Using data collected in 2011–2012 from the CHARLS national survey, we found regional geographic variations in rates of hypertension diagnosed, measured, prevalence, awareness, treatment and control among middle-aged and older adults in Chinese provinces and municipalities. According to quintiles for each category of rates we classified, several provinces and municipalities are of great concern. And ordinal logistic regressions were used to analyze the association between the multiple risk factors and quintiles of the rates of hypertension prevalence and the rates of hypertension control, respectively.

By map visualization, our results show that:

Firstly, high hypertension prevalence rates but low hypertension awareness, treatment and control rates: Guizhou. Findings from our study indicate that 56.3% of the residents in Guizhou have hypertension, and of these individuals, 37.3%, the lowest awareness rates among the provinces and municipalities, are aware of their hypertensive status. Only 21.1%, also the lowest treatment rates, of the residents who are aware of their conditions are currently taking medication for high blood pressure, only less 15% of hypertensive patients have their blood pressure below the SBP/DBP of 140/90 mm Hg. Guizhou is an under-developing provinces, and has poor health infrastructure, low educational level and lack of chronic disease prevention, which may contribute to low hypertension treatment and control rates^35^.

Secondly, high hypertension prevalence rates but high hypertension awareness and treatment rates: Shanghai and Beijing. Hypertension prevalence, awareness and treatment rates are highest in Shanghai and Beijing. But Shanghai have the highest diagnosed and measured rates among these provinces and municipalities the same as its prevalence rates. 65.0% of the residents in Shanghai have hypertension, 87.8% of the hypertensive patients are aware of their hypertensive status. Although 80.0%, the highest hypertension treatment rates, of the residents who are aware of their conditions are currently taking medication for high blood pressure, 1 in 5 of hypertensive have their blood pressure below the SBP/DBP of 140/90 mm Hg. Compared to Shanghai, Beijing have the similar hypertension prevalence, awareness and treatment rates, 57.5%, 88.6% and 77.5%, respectively, but the hypertensive patients in Beijing are controlled better, which may be lower measured rates are more easily to control. Those who with hypertension keep their blood pressure well controlled in Beijing. The two municipalities are developed regions, and have perfect health care facilities, better high blood pressure prevention work and better quality education, which may contribute to hypertensive patients well understand their conditions. Because of this, patients with high blood pressure may be positively receiving anti-hypertensive medication to control their high blood pressure.

Thirdly, low hypertension awareness, treatment and control rates: Guangdong and Guangxi. Guangdong is adjacent to Guangxi, so the two provinces have similar geographical, climatic conditions and lifestyle, which may contribute to very similar hypertension awareness, treatment and control rates. 37.5% of the residents in Guangdong are aware of their hypertensive status. 31.4% of the residents who are aware of their conditions are currently taking medication for high blood pressure, but only 6.1%, the lowest control rates, of hypertensive patients have their blood pressure below the SBP/DBP of 140/90 mm Hg. Compared to Guangdong, Guangxi have the similar hypertension awareness, treatment and control rates, 39.7%, 33.0% and 8.8%, respectively. Most of hypertensive patients in Guangdong and Guangxi are unaware of their hypertensive status, and not well treated and controlled, so the prevention and control of hypertension should be caused enough attention in the two provinces.

Fourthly, high prevalence rates but moderate awareness, treatment and control rates: Hebei, Inner Mongolia and Qinghai. Compared to Shanghai and Beijing, hypertension prevalence rates in Hebei, Inner Mongolia and Qinghai are also very high, but awareness, treatment and control rates are at moderate level. Prevention and control of hypertension are worse than Shanghai and Beijing, which may be due to their underdevelopment.

Finally, low hypertension prevalence rates but low hypertension awareness, treatment and control rates: Fujian, Sichuan, Hubei and Yunnan. 27.4%, the lowest hypertension prevalence rates, of the residents in Fujian have hypertension, and lowest hypertension prevalence rates are also observed in Sichuan, Hubei and Yunnan. While in the four provinces, most of hypertensive patients are unware of their hypertensive status, and not being well treated and controlled. Perhaps hypertensive patients in these provinces are much less, the residents are more likely to ignore their high blood pressure. The middle-aged and older adults have inadequate care for themselves. In addition, Hunan and Chongqing are similar to these four provinces in hypertension prevalence, but hypertensive patients are treated and controlled better than the latter.

Turning to the disparity of the prevalence of hypertension among provinces and municipalities, our results show that being of overweight or obesity, smoking, drinking, higher education and higher household income are risk factors. For the disparity of the hypertension control among provinces and municipalities, our results show that being of older population, the married, overweight or obesity, higher education and higher household income are main factors.

First, there was no difference in the prevalence of hypertension in age among the provinces, and older adults with hypertension control their high blood pressure better. This difference is likely because of higher rates of treatment with medication among older adults^16^,^36^.

Second, there was no difference in the prevalence of hypertension in marriage, and the married have better hypertension control. It may be that married people have more social support, less psychological stress and are more likely to incorporate positive behaviors for a healthier lifestyle, which contribute to long-term health^37^,^38^,^39^.

Third, the higher the level of overweight or obesity, the higher prevalence rate of hypertension, but there is better hypertension control among the overweight or obesity.

Fourth, the prevalence of hypertension is significantly higher for smokers than non-smokers, and higher for drinker than abstainer, which are consist with many studies^40^,^41^,^42^,^43^.

Fifth, specially, the prevalence of hypertension appears to increase with increasing socio-economic status as measured by level of education and household income, which have apparent impact on the effective control of hypertension.

There are still some limitations in our study. First, some provinces are not included in the CHARLS, including Tibet, Ningxia and Hainan. Second, CHARLS does not collect information on respondents’ dietary patterns, which may offer additional explanatory power. Third, only a random subsample of households was asked about the amount of time you spend on different types of physical activities, we could not adequately analyze the effects of physical activity contribute to the outcomes. However, it is useful studying the intriguing geographic distributions in hypertension diagnosed, measured, prevalence, awareness, treatment and control rates among middle-aged and older adults in China through map visualization, and explore the main factors of the disparity by ordinal logistic regression.

## Conclusion

We presented geographic distributions rates of hypertension diagnosed, measured, prevalence, awareness, treatment and control among middle-aged and older adults for 28 provinces and municipalities in China, except for Tibet, Ningxia and Hainan, and explore the main factors of the disparity. In our study, we found that among middle-aged and older adults Chinese with hypertension, there were provincial variations in diagnosed, measured, prevalence, awareness, treatment and control rates through map visualization. Notably, high hypertension prevalence but low hypertension awareness, treatment and control rates were observed Guizhou. Shanghai and Beijing had the highest hypertension prevalence rates, awareness and treatment rates, but the control rates was remarkably higher in Beijing than Shanghai. High hypertension prevalence rates were also observed in Hebei, Inner Mongolia and Qinghai. And Fujian had the lowest hypertension prevalence rates. Remarkable variations were observed among surveyed provinces and municipalities. Additional regional collaborative studies are needed to further validate these geographic variations across provinces, and further studies are required to improve risk factor identification, regionally target interventions and hypertension management in China.

## Additional Information

**How to cite this article**: Yin, M. *et al.* Geographic Distributions in Hypertension Diagnosis, Measurement, Prevalence, Awareness, Treatment and Control Rates among Middle-aged and Older Adults in China. *Sci. Rep.* **6**, 37020; doi: 10.1038/srep37020 (2016).

**Publisher’s note:** Springer Nature remains neutral with regard to jurisdictional claims in published maps and institutional affiliations.

## Figures and Tables

**Table 1:** Hypertension prevalence, characteristics of surveyed respondents, China, 2011–2012.

	% Unweighted	% Weighted
Hypertension		
Diagnosed	24.0 (23.2, 24.8)	24.2 (23.1, 25.3)
Measured	30.6 (29.8, 31.4)	31.9 (30.7, 33.1)
Prevalence	40.6 (39.8, 41.4)	41.7 (40.5, 42.9)
Awareness	59.0 (57.7, 60.3)	57.9 (55.9, 59.9)
Treatment	46.4 (45.0, 47.8)	46.4 (44.4, 48.4)
Control	24.7 (23.6, 25.8)	23.4 (22.0, 24.8)
Setting		
Urban area	37.0 (36.2, 37.8)	44.3 (43.0, 45.6)
Rural area	63.0 (62.2, 63.8)	55.7 (54.4, 57.0)
Sex		
Male	46.9 (46.0, 47.8)	46.4 (45.2, 47.6)
Female	53.1 (52.3, 53.9)	53.6 (52.3, 54.9)
Age (years)		
<50	20.7 (20.0, 21.4)	21.8 (20.7, 22.9)
50–59	34.5 (33.7, 35.3)	33.8 (32.6, 35.0)
60–69	28.4 (27.6, 29.2)	26.7 (25.7, 27.7)
70–79	13.2 (12.6, 13.8)	13.5 (12.8, 14.2)
≥80	3.2 (3.0, 3.4)	4.2 (3.8, 4.6)
Marital status		
Married or cohabiting	87.1 (86.5, 87.7)	85.4 (84.6, 86.2)
Unmarried or single	12.9 (12.4, 13.4)	14.6 (13.7, 15.5)
Education		
Illiterate	28.3 (27.6, 29.0)	27.2 (26.3, 28.1)
Primary	40.8 (40.0, 41.6)	39.8 (38.6, 41.0)
Secondary	29.1 (28.4, 29.8)	30.7 (29.5, 31.9)
College and above	1.7 (1.5, 1.9)	2.3 (1.7, 2.9)
Household income (tercile)^a^		
First tercile	32.8 (32.0, 33.6)	29.9 (29.0, 30.8)
Second tercile	34.9 (34.1, 35.7)	33.0 (31.9, 34.1)
Third tercile	32.3 (31.5, 33.1)	37.1 (35.8, 38.4)
N	13,583	13,583

^a^First tercile is the richest; Third is the poorest.

**Table 2:** Hypertension diagnosed, measured, prevalence, awareness, treatment and control rates for 28 serveyed provinces in China, 2011–2012, unweighted rates.

	Hypertension					
	Diagnosed (%)	Measured (%)	Prevalence (%)	Awareness (%)	Treatment (%)	Control (%)
Anhui	31.5 (3.3)	28.7 (3.3)	44.1 (3.7)	71.5 (5.3)	51.1 (5.5)	35.0 (5.2)
Beijing	50.6 (10.5)	30.6 (8.8)	58.8 (10.7)	86.0 (12.5)	78.0 (13.5)	48.0 (13.3)
Chongqing	21.8 (5.3)	29.3 (6.1)	37.2 (6.6)	58.6 (11.8)	38.6 (10.6)	21.4 (8.0)
Fujian	13.8 (3.0)	21.2 (3.7)	26.9 (4.0)	51.3 (9.1)	38.1 (8.5)	21.2 (6.5)
Gansu	23.2 (4.4)	27.0 (4.6)	39.5 (5.2)	58.5 (8.8)	45.5 (8.5)	31.7 (7.6)
Guangdong	16.1 ((2.5)	33.7 (3.3)	38.3 (3.4)	41.9 (5.6)	30.3 (5.1)	12.0 (3.3)
Guangxi	13.8 (2.8)	31.3 (4.1)	34.3 (4.2)	40.4 (7.3)	34.2 (6.9)	8.7 (3.5)
Guizhou	17.2 (5.1)	47.2 (7.5)	54.6 (7.7)	31.5 (8.8)	14.6 (5.9)	13.5 (5.7)
Hebei	34.1 (3.8)	36.3 (3.9)	52.0 (4.2)	65.6 (5.7)	58.2 (5.9)	30.1 (5.0)
Heilongjiang	32.1 (6.0)	25.9 (5.4)	41.5 (6.4)	77.3 (9.9)	50.0 (10.3)	37.5 ((9.4)
Henan	25.7 (2.5)	30.0 (2.6)	40.2 (2.8)	63.9 (4.5)	52.3 (4.6)	25.4 (3.8)
Hubei	18.1 (3.3)	29.8 (4.1)	36.5 (4.4)	49.7 (7.7)	28.3 (6.4)	18.2 (5.2)
Hunan	19.7 (2.8)	24.2 (3.1)	33.0 (3.4)	59.7 (6.6)	47.1 (6.5)	26.7 (5.4)
Jiangsu	28.9 (3.4)	30.2 (3.5)	44.1 (3.8)	65.6 (5.8)	52.7 (5.9)	31.5 (5.1)
Jiangxi	19.7 (2.9)	28.9 (3.3)	37.1 (3.6)	53.0 (6.2)	40.2 (5.9)	22.1 (4.7)
Jilin	28.2 (4.4)	34.0 (4.8)	42.4 (5.1)	66.7 (8.0)	57.1 (8.1)	19.7 (5.6)
Liaoning	24.0 (3.8)	31.6 (4.2)	40.9 (4.6)	58.8 (7.4)	50.3 (7.3)	22.6 (5.6)
Inner Mongolia	35.4 (3.9)	40.2 (4.1)	53.0 (4.2)	66.8 (5.6)	59.2 (5.8)	24.2 (4.6)
Qinghai	31.5 (7.4)	40.0 (8.0)	50.8 (8.6)	62.1 (12.1)	54.5 (12.0)	21.2 (8.2)
Shaanxi	25.8 (3.7)	25.8 (3.7)	38.0 (4.2)	68.0 (7.2)	52.5 (7.3)	32.0 (6.3)
Shandong	26.6 (2.5)	33.0 (2.6)	44.7 (2.8)	59.4 (4.3)	48.2 (4.2)	26.2 (3.6)
Shanghai	56.7 (17.8)	53.3 (17.4)	66.7 (18.2)	85.0 (22.3)	75.0 (22.6)	20.0 (12.2)
Shanxi	27.4 (4.1)	33.4 (4.4)	44.2 (4.7)	62.0 (7.3)	55.4 (7.2)	24.5 (5.7)
Sichuan	17.2 (2.1)	26.4 (2.5)	33.8 (2.7)	50.8 (5.0)	34.5 (4.5)	21.9 (3.8)
Tianjin	33.1 (7.2)	23.2 (6.2)	42.3 (7.9)	78.3 (12.1)	58.3 (12.7)	45.0 (12.0)
Xinjiang	23.1 (7.5)	34.1 (9.0)	40.7 (9.6)	56.8 (16.1)	43.2 (14.7)	16.2 (8.7)
Yunnan	17.1 (2.4)	32.2 (3.0)	38.3 (3.2)	44.6 (5.3)	29.2 (4.6)	16.0 (3.6)
Zhejiang	29.9 (4.3)	28.1 (4.2)	42.3 (4.8)	70.6 (7.3)	62.9 (7.5)	33.5 (6.7)

Surveyed provinces not including Tibet, Ningxia and Hainan in mainland china.

**Table 3:** Hypertension diagnosed, measured, prevalence, awareness, treatment and control rates for 28 serveyed provinces in China, 2011–2012, weighted rates.

	Hypertension					
	Diagnosed (%)	Measured (%)	Prevalence (%)	Awareness (%)	Treatment (%)	Control (%)
Anhui	31.3 (3.8)	27.3 (3.4)	43.1 (4.1)	72.6 (5.6)	52.2 (6.2)	36.7 (6.1)
Beijing	51.0 (11.3)	28.6 (8.9)	57.5 (11.5)	88.6 (11.0)	77.5 (14.5)	50.3 (14.4)
Chongqing	22.4 (6.0)	28.3 (6.5)	36.0 (7.1)	62.1 (12.8)	39.5 (11.8)	21.3 (8.7)
Fujian	13.1 (3.1)	22.0 (4.1)	27.4 (4.5)	47.8 (9.9)	34.7 (8.7)	19.6 (6.6)
Gansu	22.7 (4.7)	28.4 (5.3)	40.6 (6.0)	55.9 (9.6)	44.5 (9.1)	30.2 (7.9)
Guangdong	15.8 (5.8)	39.5 (8.0)	42.0 (8.0)	37.5 (12.6)	31.4 (12.2)	6.1 (2.3)
Guangxi	14.1 (3.2)	32.4 (4.5)	35.5 (4.6)	39.7 (8.0)	33.0 (7.4)	8.8 (3.8)
Guizhou	21.0 (6.8)	47.9 (8.3)	56.3 (8.4)	37.3 (11.0)	21.1 (9.2)	14.9 (6.8)
Hebei	33.5 (4.2)	38.7 (4.6)	53.9 (4.6)	62.1 (6.8)	57.2 (6.7)	28.3 (5.5)
Heilongjiang	32.4 (6.4)	27.8 (6.3)	41.8 (7.1)	77.6 (10.9)	51.5 (11.3)	33.5 (9.5)
Henan	26.1 (2.7)	31.1 (3.0)	41.2 (3.2)	63.4 (5.3)	52.3 (5.2)	24.6 ((4.0)
Hubei	18.7 (3.9)	31.0 (4.7)	37.8 (4.9)	49.5 (8.6)	26.5 (6.6)	17.9 (5.6)
Hunan	20.7 (3.3)	24.6 (3.6)	33.6 (4.1)	61.8 (7.4)	48.9 (7.5)	26.7 (6.1)
Jiangsu	29.6 (3.9)	30.9 (3.9)	45.3 (4.4)	65.5 (6.8)	51.8 (6.8)	31.7 (6.1)
Jiangxi	20.4 (3.2)	27.4 (3.6)	36.6 (4.0)	55.9 (6.9)	42.8 (6.8)	25.2 (5.8)
Jilin	29.3 (5.0)	32.0 (4.9)	41.3 (5.5)	70.9 (8.0)	60.9 (8.5)	22.5 (6.9)
Liaoning	27.9 (5.0)	34.7 (5.2)	46.0 (5.6)	60.8 (8.8)	54.3 (8.8)	24.4 (7.2)
Inner Mongolia	33.9 (4.5)	38.8 (4.6)	51.7 (5.0)	65.6 (6.5)	58.6 (6.5)	25.1 (5.6)
Qinghai	31.6 (7.5)	39.3 (8.1)	50.5 (8.6)	62.6 (12.3)	54.9 (12.2)	22.3 (8.6)
Shaanxi	31.9 (5.3)	31.8 (5.4)	45.6 (5.6)	69.9 (9.3)	61.0 (9.3)	30.4 (7.8)
Shandong	25.4 (3.1)	34.1 (4.9)	45.1 (4.9)	56.4 (8.4)	44.7 (6.9)	24.5 (4.5)
Shanghai	57.1 (21.8)	51.9 (21.3)	65.0 (22.7)	87.8 (21.4)	80.0 (23.1)	20.2 (13.0)
Shanxi	27.8 (4.6)	35.2 (5.0)	45.2 (5.2)	61.6 (8.2)	56.4 (8.2)	22.1 (5.6)
Sichuan	16.3 (2.1)	26.3 (2.7)	33.2 (2.9)	49.2 (5.5)	33.1 (4.9)	20.9 (4.1)
Tianjin	33.1 (7.5)	25.5 (6.9)	43.2 (8.3)	76.6 (13.7)	55.3 (13.4)	40.9 (11.9)
Xinjiang	28.0 (9.3)	40.6 (10.6)	47.1 (10.9)	59.4 (16.9)	43.5 (15.7)	13.8 (7.9)
Yunnan	16.9 (2.6)	32.9 (3.4)	38.7 (3.5)	43.6 (5.7)	28.7 (4.9)	15.0 (3.6)
Zhejiang	31.3 (5.5)	29.6 (5.4)	45.2 (6.2)	69.4 (9.5)	64.2 (9.4)	34.5 (8.4)

Surveyed provinces not including Tibet, Ningxia and Hainan in mainland china.

**Table 4:** Ordinal Logistic Regression Results and the Risk Factors of the hypertension prevalence in China: 45+ years old.

Variables	Coefficient	Standard error	t value	p value	Odds ratio	95% CI
Individual characteristics						
Age	0.000	0.002	0.029	0.977	1.00	1.00–1.00
Male	−0.012	0.045	−0.278	0.781	0.99	0.90–1.08
Married	0.037	0.049	0.754	0.451	1.04	0.94–1.14
Behavioral health						
BMI category						
Normal weight						
Overweight	0.363	0.036	10.155	0.000***	1.44	1.34–1.54
Obesity	0.739	0.072	10.322	0.000***	2.09	1.82–2.41
Smoking						
Non-smoker						
Past smoker	0.196	0.062	3.177	0.001**	1.22	1.08–1.37
Current smoker	0.182	0.045	4.062	0.000***	1.20	1.10–1.31
Drinking						
Abstainer						
Current drinker	0.259	0.039	6.569	0.000***	1.30	1.20–1.40
Socioeconomic gradient						
Education						
Illiterate						
Primary	−0.098	0.040	−2.457	0.014*	0.91	0.84–0.98
Secondary	0.238	0.047	5.109	0.000***	1.27	1.16–1.39
College and above	0.585	0.128	4.569	0.000***	1.79	1.40–2.31
Adjusted household income						
First tercile						
Second tercile	0.025	0.038	0.658	0.511	1.03	0.95–1.10
Third tercile	0.118	0.040	2.936	0.003**	1.13	1.04–1.22
**Intercepts**	**Value**	**Standard error**				
1|2	−0.795	0.135				
2|3	0.221	0.135				
3|4	1.326	0.136				
4|5	2.383	0.137				

Dependent variable: The hypertension prevalence was divided into quintiles. (i.e., the respondents were split into five groups of similar size at 20%, 40%, 60%, and 80% after ranking all the provinces and municipalities by the rates of hypertension prevalence). 0–20% quintile = 1, 20–40% quintile = 2, 40–60% quintile = 3, 60–80% quintile = 4, 80–100% quintile = 5. *p < 0.05, **p < 0.01, ***p < 0.001.

**Table 5:** Ordinal Logistic Regression Results and the Main Factors of hypertension control in China: 45+ years old.

Variables	Coefficient	Standard error	t value	p value	Odds ratio	95% CI
Individual characteristics						
Age	0.008	0.002	4.382	0.000***	1.01	1.00–1.01
Male	−0.016	0.045	−0.348	0.728	0.98	0.90–1.07
Married	0.177	0.049	3.607	0.000***	1.19	1.08–1.32
Behavioral health						
BMI category						
Normal weight						
Overweight	0.234	0.035	6.630	0.000***	1.26	1.18–1.36
Obesity	0.420	0.069	6.114	0.000***	1.52	1.33–1.74
Smoking						
Non-smoker						
Past smoker	0.158	0.062	2.558	0.011*	1.17	1.04–1.32
Current smoker	−0.009	0.045	−0.190	0.849	0.99	0.91–1.08
Drinking						
Abstainer						
Current drinker	0.033	0.039	0.851	0.395	1.03	0.96–1.12
Socioeconomic gradient						
Education						
Illiterate						
Primary	−0.208	0.040	−5.170	0.000***	0.81	0.75–0.88
Secondary	0.046	0.046	0.991	0.322	1.05	0.96–1.15
College and above	0.490	0.119	4.128	0.000***	1.63	1.29–2.06
Adjusted household income						
First tercile						
Second tercile	0.023	0.037	0.622	0.534	1.02	0.95–1.10
Third tercile	0.345	0.040	8.625	0.000***	1.41	1.31–1.53
**Intercepts**	**Value**	**Standard error**				
1|2	−0.616	0.136				
2|3	0.212	0.135				
3|4	1.371	0.136				
4|5	2.450	0.137				

Dependent variable: The hypertension control was divided into quintiles. (i.e., the respondents were split into five groups of similar size at 20%, 40%, 60%, and 80% after ranking all the provinces and municipalities by the rates of hypertension control). 0–20% quintile = 1, 20–40% quintile = 2, 40–60% quintile = 3, 60–80% quintile = 4, 80–100% quintile = 5. *p < 0.05, **p < 0.01, ***p < 0.001.
